# Supplementary material for: High Transmission Potential of West Nile Virus Lineage 1 for Cx. pipiens s.l. of Iran
Source: Viruses. 2020 Apr 3;12(4):397. doi: 10.3390/v12040397 (PMC7232300; doi:10.3390/v12040397)
Supplement: Supplementary file 1 [file viruses-12-00397-s001.pdf]

viruses MDPI

Supplementary Table S1. Details of the mosquitoes used in the present investigation, their SQ mean (RT-qPCR) and saliva titer (pfu/saliva).

dpi	Code of the mosquitoes	SQ Mean (RT-qPCR)	Sample type	Saliva titer (pfu/saliva)
0	1	55571.88764	Abdomen	NA
0	2	45154.53633	Abdomen	NA
0	3	62378.98617	Abdomen	NA
7	1	194474.73432	Abdomen	NA
7	2	136977.47536	Abdomen	NA
7	3	111516.23597	Abdomen	NA
7	4	601460.08626	Abdomen	NA
7	5	3600745.01418	Abdomen	NA
7	6	1395147.54063	Abdomen	NA
7	7	11547.70563	Abdomen	NA
7	8	2260006.07550	Abdomen	NA
7	9	0.00000	Abdomen	NA
7	10	118458.65836	Abdomen	NA
7	11	1079144.36707	Abdomen	NA
7	12	277107.38776	Abdomen	NA
7	13	4006841.72872	Abdomen	NA
7	14	810219.43020	Abdomen	NA
7	15	1381577.45241	Abdomen	NA
7	16	2006895.39411	Abdomen	NA
7	17	0.00000	Abdomen	NA
7	18	286839.20083	Abdomen	NA
7	19	401057.33399	Abdomen	NA
7	20	9041282.89609	Abdomen	NA
7	21	164067.99743	Abdomen	NA
7	22	9510906.77743	Abdomen	NA
7	23	0.00000	Abdomen	NA
7	24	11034335.80874	Abdomen	NA
7	25	1399.38661	Abdomen	NA
7	26	676539.36056	Abdomen	NA
7	27	1400371.16517	Abdomen	NA
7	28	1573179.84604	Abdomen	NA
7	29	21631308.73907	Abdomen	NA
14	1	0.00000	Abdomen	NA
14	2	68340883.80842	Abdomen	NA
14	3	6296708.99257	Abdomen	NA
14	4	4515646.31186	Abdomen	NA
14	5	1464.59420	Abdomen	NA
14	6	22866788.07418	Abdomen	NA
14	7	0.00000	Abdomen	NA
14	8	1004.95862	Abdomen	NA
14	9	844064.59899	Abdomen	NA
14	10	16972971.40799	Abdomen	NA
14	11	4082802.37208	Abdomen	NA
14	12	21153721.44630	Abdomen	NA
14	13	7474.83567	Abdomen	NA
14	14	53452532.12837	Abdomen	NA
14	15	1624.60163	Abdomen	NA
14	16	49427286.70608	Abdomen	NA
14	17	1116624.38770	Abdomen	NA
14	18	38106066.00757	Abdomen	NA
14	19	115211542.75102	Abdomen	NA
14	20	30496415.04052	Abdomen	NA
14	21	20175.55337	Abdomen	NA
14	22	325051.45618	Abdomen	NA
14	23	16497194.04893	Abdomen	NA
14	24	17639352.86681	Abdomen	NA
14	25	16391890.96844	Abdomen	NA
14	26	2599.51004	Abdomen	NA
14	27	3534585.11337	Abdomen	NA
14	28	3454.40770	Abdomen	NA
14	29	25265476.98450	Abdomen	NA
14	30	29466082.89145	Abdomen	NA

14	31	49596417.80783	Abdomen	NA
21	1	75982643.93058	Abdomen	NA
21	2	32896267.62299	Abdomen	NA
21	3	17605702.55848	Abdomen	NA
21	4	9524240.05744	Abdomen	NA
21	5	18734031.01044	Abdomen	NA
21	6	556.79408	Abdomen	NA
21	7	754.29954	Abdomen	NA
21	8	613.72129	Abdomen	NA
21	9	70789129.45515	Abdomen	NA
21	10	37682312.19163	Abdomen	NA
21	11	18080483.73472	Abdomen	NA
21	12	15489599.45821	Abdomen	NA
21	13	53688940.58512	Abdomen	NA
21	14	72883272.98300	Abdomen	NA
21	15	46251161.15794	Abdomen	NA
21	16	33916912.43518	Abdomen	NA
21	17	89754231.81331	Abdomen	NA
21	18	37127686.49791	Abdomen	NA
21	19	40558205.26110	Abdomen	NA
21	20	619.19339	Abdomen	NA
21	21	0.00000	Abdomen	NA
21	22	72717571.66013	Abdomen	NA
21	23	71713734.95031	Abdomen	NA
21	24	0.00000	Abdomen	NA
21	25	37741653.27284	Abdomen	NA
21	26	0.00000	Abdomen	NA
21	27	32157627.81600	Abdomen	NA
21	28	65245076.19445	Abdomen	NA
21	29	0.00000	Abdomen	NA
21	30	13401181.98695	Abdomen	NA
21	31	0.00000	Abdomen	NA
21	1	67349513.56438	Head	NA
21	2	50062192.93440	Head	NA
21	3	69064468.84096	Head	NA
21	4	29235329.23376	Head	NA
21	5	37268259.72586	Head	NA
21	6	3946.04029	Head	NA
21	7	0.00000	Head	NA
21	8	40491.55430	Head	NA
21	9	32942419.96309	Head	NA
21	10	44246080.47098	Head	NA
21	11	8641488.51333	Head	NA
21	12	22599174.45270	Head	NA
21	13	29577697.83095	Head	NA
21	14	45980776.50947	Head	NA
21	15	29904169.86367	Head	NA
21	16	42384818.45686	Head	NA
21	17	37773730.60964	Head	NA
21	18	74409397.98166	Head	NA
21	19	78918654.46148	Head	NA
21	20	0.00000	Head	NA
21	21	0.00000	Head	NA
21	22	39152588.49023	Head	NA
21	23	31897599.60575	Head	NA
21	24	0.00000	Head	NA
21	25	23131689.61730	Head	NA
21	26	0.00000	Head	NA
21	27	57063466.12809	Head	NA
21	28	46846777.40020	Head	NA
21	29	0.00000	Head	NA
21	30	41638587.93359	Head	NA
21	31	0.00000	Head	NA
21	1	NA	Saliva	360
21	2	NA	Saliva	0
21	3	NA	Saliva	130
21	4	NA	Saliva	360
21	5	NA	Saliva	40
21	6	NA	Saliva	0

21	7	NA	Saliva	0
21	8	NA	Saliva	0
21	9	NA	Saliva	30
21	10	NA	Saliva	10
21	11	NA	Saliva	0
21	12	NA	Saliva	4
21	13	NA	Saliva	4
21	14	NA	Saliva	0
21	15	NA	Saliva	10
21	16	NA	Saliva	2
21	17	NA	Saliva	20
21	18	NA	Saliva	160
21	19	NA	Saliva	10
21	20	NA	Saliva	0
21	21	NA	Saliva	0
21	22	NA	Saliva	1.33×10^5
21	23	NA	Saliva	10
21	24	NA	Saliva	0
21	25	NA	Saliva	180
21	26	NA	Saliva	0
21	27	NA	Saliva	100
21	28	NA	Saliva	20
21	29	NA	Saliva	0
21	30	NA	Saliva	0
21	31	NA	Saliva	0
